# Human Chagas Disease and Migration in the Context of Globalization: Some Particular Aspects

**DOI:** 10.1155/2013/789758

**Published:** 2013-03-30

**Authors:** João Carlos Pinto Dias

**Affiliations:** René Rachou Research Center, Oswaldo Cruz Foundation, Avenida Augusto de Lima 1.715, Barro Preto, 30.190-002 Belo Horizonte, MG, Brazil

## Abstract

Human Chagas disease originated in Latin America, being spread around the world in relation with multiple bioecological, sociocultural, and political factors. The process of the disease production and dispersion is discussed, emphasizing the human migration and correlated aspects, in the context of globalization. Positive and negative consequences concern the future of this trypanosomiasis, mainly in terms of the ecologic and sociopolitical characteristics of the endemic and nonendemic countries.

## 1. Introduction

In a broad sense, human Chagas Disease (HCD) reflects the bioecological, historic, and social situations of Latin America (LA), presenting a remarkable medical and social impact in the region [1, 2]. In its origin the disease was restricted to rural areas of LA, with a socio-political context strongly marked by poverty, human movements, and very weak productive relations. Emerging from a sylvatic cycle of the protozoan *Trypanosoma (Schizotrypanum) cruzi *(*T. cruzi*), HCD progressively became engaged with the political context of endemic countries and with a low standard of living of their population [3–5]. Following the circulation of the parasite during several thousands of years amongst different mammalian reservoirs and invertebrate vectors, in different ecological sceneries of the continent, the infection reached human beings in the so-called domestic cycle, resulting from man invasion of the natural environment. People migration, very poor conditions of life, and multiple situations of anthropic activities have been considered as the most important epidemiological factors of HCD spreading in LA [6–9]. In the last century, the progressive urbanization and the intensive migration of infected individuals increased the risk of HCD transmitted by blood transfusion and congenital route also in nonendemic regions [5, 6, 10]. In the present work, a general discussion about the role and the main antecedents of migration and other epidemiological aspects of HCD is intended, in the context of globalization.

## 2. The Medical Impact and the Fight against HCD

Until the last century, HCD affected primordially rural people living in poor dwellings colonized by the vector insect. It has been extremely impacting disease, with expressive mortality among children in its acute phase and severe heart lesions in about 10–20% of chronic adult patients. Its medical and social burdens involve mortality, high hospital and social costs, absenteeism, and labor incapacity [1, 11–13]. For recent years the prevalence of 8-9 million of infected individuals in LA has been estimated, with an expected mortality between 23 and 45 thousand individuals per year, depending on the source of information [14, 15]. The population under the risk of transmission is calculated to be about 30–40 million [10, 16]. The most effective strategies to prevent HCD consist in vector chemical control, housing improvement, and rigorous serologic screening of blood donors. There is no available strategy to prevent congenital and oral transmitted HCD, but early diagnosis and specific treatment of infected individuals are strongly recommended [10]. The specific treatment with the currently available drugs (Nifurtimox and Benznidazole) is more effective in acute cases, young people, and cases of recent transmission, but now the treatment of indeterminate and initial chronic cases is also being suggested [17–19]. The correct and permanent medical assistance in chronic cases is considered fundamental to improve the quality and quantity of life of infected individuals [20–22]. In the last thirty years, control programs, large scale screening of blood donors, medical care, and the emptying of rural population have contributed to minimizing the social burden of HCD [1, 2]. Nevertheless, it is a mistake to imagine that HCD has been completely conquered; in some countries, regular control programs have not been implemented and, in others, epidemiological surveillance is under the risk of being relaxed. By another side, congenital and oral transmitted cases still exist [1]. The basic challenges of HCD for the next two decades consist of (a) launching adequate programs in those endemic countries without control activities, (b) improving and sustaining the existing programs, mainly in terms of epidemiological surveillance, and (c) implementing and improving adequate medical care for the already infected individuals [16, 23–25].

## 3. Globalization in Latin America

Social and political changes of the whole society have been very expressive in the last decades, following the process of globalization, all around the world. Among other factors such as the increasing of human migration, globalization has produced an economical effect tremendously unequal among different countries and social groups, as a consequence of economical speculations, international and supranational competition and political affairs [24, 26].

Regarding the overcoming of neglected diseases, Dr. Margareth Chan, Director-General of WHO, said very clearly that “efforts to control neglected tropical diseases constitutes a pro-poor strategy on a grand scale. The logic has changed: instead of waiting for these diseases to gradually disappear as countries develop and living conditions improve, a deliberate effort to make them disappear is now viewed as a route to poverty alleviation that can itself spur socioeconomic development” [16]. In other words, the overcoming of health problems such as HCD depends on the empowerment of state structures and of social commitment of rich and poor countries [3, 24]. Usually, the logic of globalization based on market economy has deeply affected the developing countries, reinforcing social inequalities and making the possibilities of social upgrade for marginal populations very hard [5]. As Professor Aluizio Prata said some years ago, globalization implies domestic deregulation, commercial liberalization, and privatization by means of foreign and volatile capitals, resulting in progressive social inequalities and unstable economy, generating several and complex influences in health sector (Prata, A.R., 2003. Conference about Tropical Medicine in Brazil. Congress of the Brazilian Society of Tropical Medicine. Belém, Pará, Brazil, February, 2003. A*pud* Dias (2007)). Neoliberalism, a main axis of globalization, represents a movement to benefit the great world potencies and the multinational enterprises. Poor countries usually suffer with neoliberal policies, in terms of underemployment, low salaries, inequity, and strong dependency of international capitals and of the global market (http://wikimediafoundation.org/, access in June 27, 2011). Particularly, this situation used to be responsible for the intensive migration process all around the world. Regarding LA, after two decades of Neoliberalism, the World Bank evaluation in 2005 considered that the results (in terms of economic grow) remained much lower than it was formerly awaited [24, 26].

## 4. Globalization and Chagas Disease

**Figure 1:**
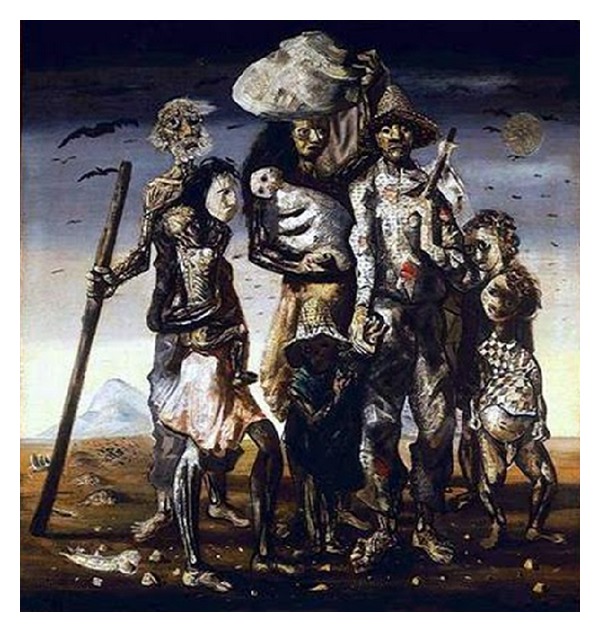
*Rural retirement*. Picture by Candido Portinari (Brazil) *In *ngeladohs.blogspot.com (accessed in Sept. 12, 2012).

In the poor endemic regions of LA, the impact of globalization has been considerable in terms of HCD epidemiology, management, and prevention [27, 28]. The increasing of migration and the progressive changes in rural economy modified the epidemiological patterns of the disease, mainly in terms of its transmission and medical attention. For instance, following the reduction of demographic densities in rural endemic areas, a minor vector infestation can be considered as a “positive” effect. In addition, several areas have been modernized in terms of housing and production aspects; it means a radical change from the classical subsistence way of life to an agroindustrial and large scale economy [7, 24]. By another angle, the expansion of agriculture frontier in some places made possible the spreading of infected individuals and even of vectors [29].  Figure 1 represents the dramatic migration of rural poor people in Brazil, leaving their native land in search of survival abroad. 

## 5. The Global Market, the Role of the State, and HCD

Regarding the social and political evolution of LA, HCD can be considered a reflex of the regional history, particularly in terms of equity and production dealings. By the side of the infected individuals, globalization and market implications produced bad and good results, spreading the disease and improving the conditions for medical access. In general, the market related with HCD is very weak; the poor chagasic individuals usually depend on public health, especially in poor endemic countries. The main commercial profits (relatively low) concern laboratory diagnostic reagents and insecticides [30]. In addition, private enterprises do not always rely on LA governments, making social projects and the acquisition of products difficult. A correlated situation concerns insecticide prices, with high disparity, in the past, among the different countries [2, 12, 13, 24]. In this aspect, globalization rules have been beneficial, mainly after the emergence of the intergovernmental initiatives against Chagas disease, inducing the equalization of prices [1, 31]. At the side of specific treatment, the development of new drugs by private industries is not stimulant, because of the weak market and commercial risks. For instance, Roche considered its product Benznidazole as “a social drug” some years ago, giving it patent to the Brazilian Government. In terms of electoral benefits, also HCD seems to be inexpressive, because *chagasic* population usually has no political density, being unable to obtain the minimum social gains [13, 24, 28]. Concerning disease prevention, different situations have been observed in LA. First of all, vector control is based basically on insecticides and housing improvement. In this aspect, the poor communities depend almost exclusively on governmental programs, in other words, on political will. Notwithstanding, several observations and mathematical models have shown that vector control can result from the social improvement of the community, without a direct governmental intervention [13, 32]. In this aspect, the governments of LA commonly give no priority to the poorest rural populations, where residual transmission exists [16, 24]. On the positive side, two different situations were affected by globalization: (a) regarding transfusion transmitted HCD, since the 1980 decade, the proportion and the quality blood control have been highly improved, following the global demand for AIDS control [10, 14, 29]; (b) since the 1990s, following the economic and political commitments of globalization, the above mentioned “intergovernmental initiatives” were launched in LA to face HCD in terms of vector and transfusion transmissions, with technical assistance of PAHO and WHO [6, 16, 33]. In general terms, Briceño-León [12], Dias [23], and Schmunis [26] observe that such initiatives are completely integrated in the scenario of retrieval and rescue possibilities of LA, a region that looks for its identity and its better political and social expression.

## 6. Migration and HCD

It can be said that in the beginning, the spreading of HCD in Latin America was related to human movements, in parallel with progressive rural settlements and the domiciliation of triatomines [5, 8, 29]. Along the history, the total majority of infected individuals have been contaminated in infested dwellings of endemic areas [10, 12]. For instance, in Brazil, Zeni [34] studied 265 seropositive individuals in Paraná state, from whom 255 declared to have been bitten by the vector in rural houses (96,22%). In São Paulo city, Goldbaum [7] detected 232 infected urban workers; all of them immigrated from rural communities of 13 Brazilian states. As an example, the arrival of infected individuals has been registered in Beni and Pando (Amazonic regions of Bolívia), as a consequence of people immigration from endemic areas where mining and agricultural economies have failed [33, 35–37]. International migration waves in LA are also significant, for instance, from Bolivia and Paraguay to Argentina and Brazil, or from Colombia to Venezuela. In other scenery, mainly since the second half part of the last century, the movement of thousands of Latin American citizens towards North America, Europe, Asia, and Oceania was intensified, thus increasing the number of infected individuals living in nonendemic countries [2, 17, 38, 39]. In Belo Horizonte city, amongst 291 seropositive blood donors, Gontijo [40] detected 249 who certainly originate from rural localities (85,6%). This proportion, in Recife, was 95.0% [41]. In 1987, Wanderley [42] pointed out the estimate of 350,000 infected individuals living in the metropolitan area of São Paulo city (Brazil), in the great majority who immigrated from rural zones of Brazil endemic states and from Bolivia. At the Hospital of the Federal University of South Mato Grosso, Pompilio [43] found 88.3% of rural origin amongst 120 infected patients. Studying 57 infected women in Obstetric Unities of São Paulo city, Nisida et al. [44] found 55 who originate from rural endemic areas of Brazil, all of them declaring to know the insect vector.

In Rio de Janeiro city, amongst 260 infected individuals, Coura verified that all of them originated from endemic rural zones of Brazil (256 individuals), Bolivia, and Paraguay [45]. In Argentina, chagasic individuals studied in Buenos Aires have been contaminated in rural zones of different endemic provinces and of Bolivia [46]. The same rural origin is seen for infected individuals detected in Caracas, in Spain, and in USA [12, 17, 26, 39]. Even in the small towns of endemic areas, the majority of the detected cases come from rural localities, where their contamination occurred during childhood [7, 18, 22, 35].

Departing from the basic rural situation, different flows of individuals and families can be detected along the history of LA: (a) migration inside different rural areas, resulting in vector and reservoir dispersion; (b) migration from endemic to nonendemic rural areas (it is the case of thousands of families in Brazil going to Amazon region); (c) migration from rural localities to urban centres, generally being installed in peripheral sites; (d) the reverse migration from urban to rural areas; (e) the seasonal migration of rural laborers who travel periodically to work in different unskilled labor fronts such as sugar cane crops, petrol exploration, and civil constructions; and (f) migration from endemic to nonendemic countries, departing both from rural and urban situations. Basically, HCD and human migration are correlated with the social process of poor individuals moving themselves to look for better conditions of life [12, 13, 47]. In endemic areas, rural people usually have a poor and very provisional standard of life, depending on a weak familial economy and on a very unstable working situation [7]. Their houses are poor and provisional; they are not the owners of the land [7, 12, 28, 48]. Migration becomes a question of opportunity and survival. In strict correspondence with globalization, the rural-urban migration was caused by several changes in the productive system, specially industrialization and weakening of the traditional rural economy, a continuous process that is rarely planned or assisted; it emerges as a consequence of the adverse social and economic conditions of the original place [7, 46]. As said by a rural woman in Minas Gerais, Brazil, all the family must be engaged in very poor agricultural activities, first of all to eat and then to sell the rest: “All of us, here, depend of the hoe, of the force of our arms…” and “I dream with health, with a good work… When we are bog down in a hole we dream to leave it… Our husbands must often to leave us, going to São Paulo to earn some money in sugar cane harvesting or in civil constructions…” [48]. Definitive rural emigration generally deals with the process of urbanization, basically resulting in urban peripheral settlements. When these settlements reproduce socially and ecologically the rural environment, housing infestation by means of passively carried triatomines can occur, as it was detected in Cochabamba, Sucre, Tupiza (Bolivia), in Guayaquil (Ecuador), in Tegucigalpa (Honduras), and so forth [35, 37].

Sosa [47] analyzed the migration process in the province of Tucumán, Argentina, in recent years, detecting that the total majority of chagasic individuals left their rural regions ad moved to urban centers of the country because of the lack of employment, due to extensive and semi-industrial sugar cane activities implemented all over the province. Similar situation can be observed in several parts of LA such as San Juan (Argentina), Cochabamba (Bolivia), rural areas of Venezuela, and Jequitinhonha Valley (Brazil) [10, 14, 49].

Analyzing rural emigration in endemic areas, several authors agree that its basic cause is a complex process involving the failure of the classic familial agriculture, the boom of the urban-industrial model, and the new agroindustrial perspectives for massive production. In addition, the lack of a social policy concerning agriculture in Latin America in the last century also stimulated the rural emigration: no prices, no technical assistance, and no proportion between efforts and results [13, 29, 30]. For a long period, the urban reality scared rural immigrants, generally illiterate and not able to work except by means of their physical and few differentiated forces. In 1983, studying chagasic individuals living in Belo Horizonte who immigrated from rural areas, Gontijo verified that the majority of them came to the city pursuing better working conditions, but also declaring their immense desire to go back someday [40]. The same was seen by Dias [18] in the small town of Bambui, Brazil, where all the 87 infected patients followed by 52 years since the acute phase changed from rural to urban places of residence, in order to get better work, health care, and education. The “urbanization” of HCD has been occurring all over LA, mainly after the second half part of the century XX. In the last decades, the disease reached urban areas and nonendemic countries, becoming a new medical and social problem [12, 26, 50]. Only in Brazil, it has been calculated that at least 75% of “chagasic” individuals are now living in urban spaces, a proportion that seems to be lower in other countries such as Bolivia, Guatemala, and Paraguay. In Argentina, the majority of infected individuals are living in the Great Buenos Aires [25, 37, 47, 49]. The presence of infected individuals in urban centres increased the demand for medical and social security assistance, also conveying the risk of congenital, accidental and transfusional transmission [10, 26, 29, 39, 50]. The access to medical attention certainly has been improved in the urban context, particularly in terms of more complex interventions, mostly depending on public health sector. The spreading of HCD to nonendemic countries has been studied by Schmunis, Albajar-Viñas, Storino, Zeledón, and others, receiving particular attention by WHO expert groups [2, 12, 16, 19, 26, 39].

## 7. International Flows to Nonendemic Countries

**Figure 2:**
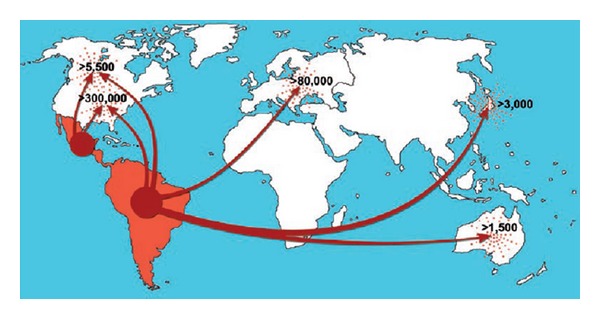
Migration routes from Latin America and estimation of the total number of infected individuals in nonendemic countries.

The general situation concerns the migration of infected people from Latin America, particularly since the end of the XIX century. Many authors have been dedicated to this subject, the consensus being that the basic cause involves a survival strategy of poor population in search of employment and better living conditions in more developed countries [12, 14, 19, 26, 39, 50–52]. The principal destination has been to USA, followed by European countries, chiefly Spain (Figure 2 [50]).

Several considerations have been made about this important theme, involving complex social and epidemiological aspects [10, 19, 50]. Concerning the risk of disease transmission, the main possibilities deal with the congenital and transfusion routes, followed by some cases involving organ transplantation. Vector transmission seems to be improbable, in spite of the eventual detection of housing invasion by *T. rubrofasciata* and *Linshcosteus sp. *in Southern Asia [25, 39, 51]. The main preoccupation concerns the demand for medical attention and social security, a problem which has been highly complicated because of the clandestine situation of thousands of LA migrants and the lack of medical expertise in nonendemic countries [17, 50, 52]. In these countries, a network of specialized centers in HCD management should be implemented, aiming to produce standards for disease treatment and control [52].

## 8. Correlated Aspects Involving Human Movements and the Dispersion of HCD in Endemic Regions

Other different situations must be focused on, not only those related to people displacement. Vectors and reservoirs are also in constant movement, being implied in the whole process of *T. cruzi* dispersion. Climatic and other ecologic factors, but mainly anthropic actions, are naturally involved in such movements. Regarding triatomine vectors, different ecotopes and domiciliation (colonization of human dwellings) depend chiefly on the species and ecologic situations [5, 10, 14, 36, 53]. Among more than 140 species registered, no more than 15 or 20 have the capacity to colonize persistently human dwellings. Adult individuals of some few wild species can eventually invade human dwellings and transmit HCD, independently of colonization, generally attracted by artificial light or searching for food [5, 38]. Originally, all the vectors of HCD were living in sylvatic ambience, associated with birds, small mammals, several amphibian, and reptile species. Human beings entered into this sylvatic cycle much later, modifying the landscape and offering new and appropriate situations to wild vectors, mainly a poor house and an abundant feeding source [10, 35]. The classical example of HCD is its expansion in Bolivian Valleys since the XVII century when the Inca People became socially stable by developing cattle and agriculture activities, attracting wild *Triatoma infestans* to their stone and mud made houses. Once colonized, the species became more and more adapted to human houses, being able to produce enormous indoors colonies. From the first focus to neighbour houses, the vector is able to disperse by means of active or passive migration. From Bolivia, *T. infestans* reached Chile, Peru, Argentina, Uruguay, and Paraguay, mainly at the expense of human displacements due to agriculture expansion and internal wars [35, 37, 38].

The history of *T. infestans* spreading in Brazil since the XIX Century is emblematic, well studied by Silva [8]. The expansion of the international market of coffee induced a tremendous boom of its cultivation, first in São Paulo State, attracting a great amount of migrants coming from other Brazilian states and other countries such as Italy and Japan. Coffee is considered the main reason for S. Paulo development and posterior industrialization. For long years, all the production had to be carried from the farming to the Santos harbour by means of equine transportation. Railways only were implemented in the last decades of 1800, also being involved with triatomine dispersion. Mules should be imported from the Rio Grande do Sul state, a traditional producer of horses, at that time already infested by *T. infestans*, originated from Paraguay and Argentina (Jesuit Missions) and from Uruguay (regular commerce). The troops carried passively the triatomines to S. Paulo during more than 80 years, chiefly arriving in the Sorocaba region, which became the epicenter of *T. infestans* dispersion in S. Paulo. The process of infestation was progressive and continuous, in direction to the west of the state, as well as to the states of Paraná and Minas Gerais, following the expansion of the coffee frontier. Coffee, the so-called green gold, became in São Paulo the basis of a new economic era, impelling immigration, deforestation, urbanization, and industrialization [8, 35, 38]. Later on, with the reverse migration of north-eastern workers, the species has been expanded to Bahia, Pernambuco, and Piaui states [52].

Another example refers to *Triatoma dimidiata *being carried passively from wild ecotopes of Costa Rica to human urban and peri urban dwellings by means of firewood transportation, a process that was being reduced when the population changed fire wood cooking to petrol gas and electricity [54].

Passive migration and active displacements of the vector are present in several other reports, including at the international level. For instance, *R. prolixus *seems to have a very complex story of its spreading from Venezuela to Mexico and Central America, involving an international scientific interchange of insect collection (Venezuela > France > El Salvador), the sea commerce between Central and South America, and even, possibly, the dispersion of eggs and nymphs, carried passively in birds migration [55]. Triatomine bugs have been identified outside America in parts of Africa, Middle East, Southeast Asia, and the Western Pacific. Current theories indicate that triatomines detected in Southeast Asia have probably derived from American species passively carried to seaports by sailing ships since the sixteenth century [38, 55]. The construction of railways and roadways has also played a significant role in the dispersion of domestic vectors in endemic countries [28, 38, 51, 54].

*Natural reservoirs*. In parallel with vectors movement, mammalian reservoirs and their mobilization in the vicinity of human houses play an important role in HCD epidemiology, particularly the so-called synanthropic reservoirs such as the opossums [5, 10, 14]. Besides being a natural source of sylvatic *T. cruzi* strains, wild reservoirs can introduce the parasite in domestic cycle, when they get closer to human dwellings harboured by triatomines. Such reservoir movements many times are a natural consequence of human intervention in natural ecotopes, chiefly when their natural shelters and food sources are destroyed [5, 14, 53]. In general terms, deforestation and massive monocultures have been appointed as the main anthropic activities in natural environments, with direct influence on reservoir and vector mobility. In terms of domestic reservoirs, the ancient culture of rural families to keep dogs, chicken, cats, guinea pigs, rabbits, and so forth very close to the house must be considered one of the most important factors to attract and to maintain domestic triatomine colonies, as well as to maintain (the mammalian ones) the cycle of the parasite [5, 29, 36].

## 9. The Role of the Remaining Sylvatic Cycle of *T. cruzi*

As a consequence of urbanization and control programs, the wild cycles of the parasite play a particular role in the maintenance of HCD. It is expectable that with the decreasing of domestic cycles in endemic areas, the major risks of HCD incidence will depend on sylvatic triatomines and wild trypanosome populations in the vicinity of susceptible human beings [5, 53]. The enzootic cycle of *T. cruzi* also has some implications for globalization. Human intensive movements and progressive modifications of sylvatic ambient (macroprojects considering deforestation, monocultures, cattle, and the extensive use of pesticides) are clearly changing the general landscape since America discovery. Particularly the emergence of several acute cases of oral transmitted HCD has been important, especially in Amazon region, basically dependent on the contamination of a series of meals of *T. cruzi* originated from wild triatomines [29, 33, 49]. Nevertheless, in the next decades, domestic cycles of HCD tend to remain in those more isolated and poor rural zones, with lower taxes of social and ambient changes [36, 53, 54]. The future will be marked by the progressive reduction of some classical species such as *T. infestans *and* R. prolixus*, besides a residual peridomestic infestation by ubiquist species (*T. dimidiata, T. pseudomaculata, *and* T. brasiliensis*, etc.). By another way, due to anthropic affairs, wild species such as *P. geniculatus, R. pictipes, T. rubrofasciata, *and* T. picturata* could occasionally invade human dwellings, eventually establishing little colonies and being able to transmit HCD [10, 38, 55]. Probably, in the future, anthropic actions and globalization will be much more associated with the enfeeblement and focalization of the sylvatic cycle of *T. cruzi* than with its exacerbation [23, 29, 56]. Several examples can be remembered, showing strong linkages between HCD and globalization mainly involving the evolution of the productive system, spatial occupation, and human movements, such as [26, 30, 50] the following:

- deforestation resulting from a strong wood market and the expansion of agroindustries in endemic areas;
- the extensive use of pesticides in agroindustrial projects;
- the expansion of the use of electricity and industrial machinery, interfering with wild triatomine behavior and influencing the rural demography;
- the progressive reduction of mammal reservoirs of the parasite, resulting from deforestation, pesticides, and extensive monocultures;
- progressive changes in the productive model, reinforcing capitalist agroindustries in detriment of the classical strategy of family subsistence;
- in the same logic, the dominant market and large scale economy will overlap the classical microeconomies;
- the modernization of agriculture, specially by means of automatic tools, robotics, and housing improvement, expulsing poor familial economies and hampering triatomine domestic colonization.

## 10. A Very Critical Point: The Medical Management Chagas Disease in a Globalized World

Presently, all over the world, the increasing life expectancy has been a great tendency of the population, resulting from better medical and social assistance. The medical management of HCD requires new knowledge and practices in terms of disease physiopathology and of the superposition of several other medical problems occurring chiefly in higher age groups, such as hypertension, diabetes, coronary diseases, Parkinsonism and physiological denervation. In such a scenery, the medical management of HCD involves three major challenges, highly depending on the political and social organization all around the world [17, 21, 22, 46, 52, 57]:

- the improvement of medical expertise for HCD management in chronic cases all over the world, mainly in terms of the primary health-care level; in corollary, the improvement of drugs and other medical proceedings is highly desirable, considering the elder patients and the superposition of other chronic and degenerative diseases;
- the betterment of medical and social security systems in order to assure adequate access and coverage for all infected individuals;
- ensuring political and administrative conditions to maintain at least two or three decades more the medical expertise able to manage adequately the infected individuals.

Other predictable situations could be emphasized for HCD management at the medium term, all of them being correlated with globalization. First of all, globalization has been a strong stimulus for the advance of medical security enterprises, able to increase the access of a progressively higher number of infected people to medical attention. The specific treatment is another important question, because it has been more and more indicated for chronic patients, trying to minimize and/or to prevent severe clinical conditions, specially advanced heart disease and sudden death. The basic problems considering this subject lie in diagnosis access, medical expertise, and drug availability, besides a good treatment adherence [2, 15, 17, 20, 57]. In chronological terms, the best moment to improve specific treatment for chronic cases of HCD has been estimated from now until one or two decades more, a time when the number of young infected individuals is still high. After 2020, with the progression of transmission control and the natural aging of infected people, the demand for specific treatment will decrease significantly. In pragmatic terms, globalization tends to facilitate specific treatment, bettering the availability of drugs around the world, chiefly with the assistance of health and humanitarian institutions such as WHO, PAHO, DNDI, and MSF [19, 21, 51].

## 11. The Future

It is admitted that HCD and other so-called neglected diseases have received and will continue to receive strong influences of the globalization process. Poor, isolated, and marginal areas in LA will continue to exist, as remaining foci of disease prevalence and active transmission. Likewise, the majority of the *chagasic* individuals will continue to be poor, illiterate, and socially excluded. National and international migration certainly will continue, spreading infected individuals in urban spaces all over the world during two or three more decades. The overcoming of HCD will depend on the public sectors (i.e., on political will) and on the reduction of inequity. As a correlated issue, considering developed nonendemic countries such as Canada, the social situation of poor immigrated individuals usually involves personal constraints and severe consequents for their health, in terms of disease management and work safety [58]. Coming back to trypanosomiasis, at the side of universal macropolicies, a recent WHO document stated that, “sustaining the progress made in controlling Chagas disease will depend on political commitment and the retention of public health resources. Resolution WHA 63.20, adopted by the Sixty-third World Health Assembly in May 2010, urges Member States where the disease is both endemic and nonendemic to control all transmission routes (namely vectors, transfusion, organ transplantation and vertical and oral routes) and to integrate the care of patients with all clinical forma of the disease into primary health-care services. WHO has been requested to facilitate networking at the global level and to reinforce regional and national capacities on strengthening global epidemiological surveillance of the disease… to advance intersectoral efforts and collaboration; and to support the mobilization of national and international public and private financial and human resources towards the achievement of these goals” [19].

In terms of political strategy, macropolicies and program organization are points to remain in the agenda of WHO and of the governments of endemic countries by the next two or three decades, keeping alive the interest and the priority of HCD and its control. National and regional programs must be adapted to the decentralization of health systems, another universal consequence of globalization [24, 30]. New nongovernmental partners such as Mèdicins Sans Frontiéres appear to be very effective and opportune to face HCD in endemic and nonendemic countries. The recent institution of a global scientific network to face HCD by WHO Neglected Diseases Department is very opportune. For the particular case of LA, it is extremely important to keep Pan American Health Organization in the coordination of the regional intergovernmental initiatives [5, 24, 29].

## 12. Final Remarks

Chagas Disease has been a concrete and impacting social and medical problem in LA, with multiple aspects associated with social iniquity and globalization. Human migration has been highly dependent on globalization and other social processes, being responsible for disease expansion and for a new epidemiologic situation, in which medical care must be improved. In spite of different financial and political constraints, HCD has been controlled, with remaining two or three more decades of program consolidation and medical attention for all infected people. This is a particular task for LA, because the fight against HCD requires the action of the state, as the basic social provider for the poorest citizens. The main strategies to face HCD have showed to be considerably effective at the medium and long terms, depending on social improvement, transmission control, and medical attention. Considering these points, the persistence of iniquity and other negative aspects of globalization have been hard challenges to be overcome in endemic areas. With the progressive reduction of its transmission and morbidity in the last two decades, the visibility of HCD as well as its political priority tends to decrease. In addition, the emergence of other public health problems such as dengue fever, influenza, and epidemic AIDS is contributing to the deviation of human and financial resources from the existing HCD programs [5, 16, 24].

Also, the transition of health sector all over LA has been slow and complicated, in spite of its highly logical and stimulant theoretical approach. Decentralization of health services and the reduction of vertical programs have been a consequence of globalization, putting the major responsibility of medical care and control programs on peripheral governmental levels [17, 39, 51]. Contextual difficulties and inequities exist but must be overcome by a universal effort involving people, governors, and scientists effectively compromised with poor populations. On the other side, the tremendous advance of medicine must be considered, in which new drugs and new diagnostic approaches are becoming available, thus raising several possibilities to improve the medical attention to HCD. Finally, at the political context, the epidemiological results of the intergovernmental initiatives can represent and stimulate a new and positive moment in the search of the continent political coherence and self-reliance [3, 12, 23, 27].
